# Supplementary material for: Patterns of Intron Gain and Loss in Fungi
Source: PLoS Biol. 2004 Nov 30;2(12):e422. doi: 10.1371/journal.pbio.0020422 (PMC532390; doi:10.1371/journal.pbio.0020422)
Supplement: Table S1 — Also available at http://genes.mit.edu/NielsenEtAl/. (4.3 MB ZIP). [file pbio.0020422.st001.zip › NielsenEtAl/html/544.html]

AN2294.1.NCU01229.1.MG05481.1.FG02040.1


```
 CLUSTAL W (1.82) Multiple Sequence Alignments - Introns Inserted


Sequence 1: NCU01229.1	571 aa
Sequence 2: FG02040.1	572 aa
Sequence 3: MG05481.1	569 aa
Sequence 4: AN2294.1	574 aa
Alignment Length: 575 aa
Number Identitical Residues: 447 aa
Alignment Score (without introns) 19048


MG05481.1 	MSTTTVARPAA----SATLAPEGERFLRCCADIANALIEDYEAAARDPKHRQKDVNLNSL
NCU01229.1	MATTTVTVTRS--KKNANLPSENDRFLRCCADIANALIEDHEAAQNG--RPQKDINLNSL
FG02040.1 	MATATMTVTAVPTKKKPDMAPESERFLRCCADVANALIEDHEASKAG--RTTRDINLNSL
AN2294.1  	MATTTMTAPPPIKTKNSKLPPENERFMRACSDIANALIQEYESQKDP-TQPKRDINLNKL
          	*:*:*:: .   ..... :..*.:**:*.*:*:*****:::*:     .:  :*:***.*

MG05481.1 	RGKFSRKHKLKKIPPLTAIIAAVPEHYKKYILPKLIAKPIR~TSSGIAVVAVMCKPHRCP
NCU01229.1	RSKFAKKHKINNVPPLTAIIAAVPEHYKKYILPRLIAKPIR~TSSGIAVVAVMCKPHRCP
FG02040.1 	RNKLAKKHKLMNIPPLTAIIASIPEHYKKYILPKLIAKPIR~TSSGIAVVAVMCKPHRCP
AN2294.1  	RGQMAKKHSLSHQPPLTAIISAVPEHYKKYILPKLIAKPIR1TSSGIAVVAVMSKPHRCP
          	*.::::**.: : *******:::**********:******* ***********.******

MG05481.1 	HIAYTGNICVYCPGGPDSDFEYSTQSYTGYEPTSMRAIRARYDPFEQARGRVDQLKALGH
NCU01229.1	HIAYTGNICVYCPGGPDSDFEYSTQSYTGYEPTSMRAIRARYDPFEQARGRVDQLKSLGH
FG02040.1 	HIAYTGNICVYCPGGPDSDFEYSTQSYTGYEPTSMRAIRARYDPFEQARGRVDQLKSLGH
AN2294.1  	HIAYTGNICVYCPGGPDSDFEYSTQSYTGYEPTSMRAIRARYDPFEQARGRVEQIRSLGH
          	****************************************************:*:::***

MG05481.1 	SVDKVEYIIMGGTFMSLPESYRDDFISQLHNALSGYQTSNVDEAVEAGEMSNVKCVGITI
NCU01229.1	SVDKVEYIIMGGTFMSLPESYRDEFISQLHNALSGYGTLNVDEAVRSGEQSNIKCVGITI
FG02040.1 	SVDKVEYIIMGGTFMSLSESYREEFIAQLHNALSGYQTTNVDEAVEAGEMSNVKCVGITI
AN2294.1  	SVDKVEYIIMGGTFMSLPAEYRDTFVAQLHNALSGYQTDNVDEAVQAGEMSNIKCVGITI
          	*****************. .**: *::********* * ******.:** **:*******

MG05481.1 	ETRPDYCLQPHLSSMLRYGCTRLEIGVQSLYEDVARDTNRGHTVAAVAETFCLAKDAGFK
NCU01229.1	ETRPDYCLQPHLSSMLRYGCTRLEIGVQSLYEDVARDTNRGHTVAAVAETFCLAKDAGFK
FG02040.1 	ETRPDYCLQPHLSDMLRYGCTRLEVGVQSLYEDVARDSNRGHTVAAVAETFCLAKDAGYK
AN2294.1  	ETRPDYCLDTHLSSMLRYGCTRLEIGVQSLYEDVARDTNRGHTVAAVAETFKLAKDAGFK
          	********:.***.**********:************:************* ******:*

MG05481.1 	VVSHMMPDLPNVGMERDVDQFREYFENPAFRTDGLKIYPTLVIRGTGLYELWRTGRYQNY
NCU01229.1	VVSHMMPDLPNVGMERDLDQFREYFENPAFRTDGLKIYPTLVIRGTGLYELWRTGRYQNY
FG02040.1 	VVSHMMPDLPNVGMERDIDQFREYFENPAFRTDGLKIYPTLVIRGTGLYELWRTGRYQNY
AN2294.1  	VVSHMMPDLPNVGMERDLFQFEEYFENPAFRTDGLKIYPTLVIRGTGLYELWRTGRYKNY
          	*****************: **.***********************************:**

MG05481.1 	TPNGLIDLVARILALVPPWTRIYRVQRDIPMPLVSSGVENGNLRELALARMKDFGTTCRD
NCU01229.1	TPNGLIDLVARIMALVPPWTRIYRVQRDIPMPLVTSGVENGNLRELALARMKDFGTTCRD
FG02040.1 	TPNGLIDLVARIMALIPPWTRIYRVQRDIPMPLVTSGVENGNLRELALARMKDFGTTCRD
AN2294.1  	TPNALIDLVARILALVPPWTRIYRVQRDIPMPLVTSGVENGNLRELALARMKDFGTTCRD
          	***.********:**:******************:*************************

MG05481.1 	VRTREVGINEVKHKIRPNQVELVRRDYVANGGWETFLAYEDPKQDILIGLLRLRKCTEKY
NCU01229.1	VRTREVGVNEVKNKIRPNQIELVRRDYTANGGWETFLAYEDPKQDILVGLLRLRKCSEKY
FG02040.1 	VRTREVGVNEIKNKIRPNQIELVRRDYVANGGWETFLAYEDPKQDILVALLRLRKCTEKY
AN2294.1  	VRTREVGINEVKNKIRPSQVELIRRDYTANGGWETFLAYEDPKQDILIGLLRLRKCSPTH
          	*******:**:*:****.*:**:****.*******************:.*******: .:

MG05481.1 	TFREELTGQPTSLVRELHVYGTAVPVHARDPKKFQHQGFGTLLMEEAERIARDEHGSEKI
NCU01229.1	TFREELVGQPTSLVRELHVYGMAVPLHVRDPRKFQHQGFGTLLMEEAERIARDEHGSVKI
FG02040.1 	TYREELIGQPTSMVRELHVYGTAVPVHARDPRKFQHQGFGTLLMEEAERIAIEEHGSDKI
AN2294.1  	TFRPEFTGQQTSIIRELHVYGSAVPLHGRDPRKFQHLGFGTLLMEEAERIAREEHGSTKI
          	*:* *: ** **::******* ***:* ***:**** ************** :**** **

MG05481.1 	SVISGVGVRSYYKKLGYWLDGPYMSKWLDGRPGN--
NCU01229.1	SVISGVGVRSYYRKLGYWLDGPYMSKWLDGRPGPEE
FG02040.1 	SVISGVGVRSYYKKLGFWLDGPYMSKWLDGREQPE-
AN2294.1  	SVISGVGVRSYYARLGYSLDGPYMSKMLEPIEGGES
          	************ :**: ******** *:     ..
```
